# Remote Check in Cochlear Implant Follow-Up: An Exploratory Comparison with Conventional Audiological Measurements

**DOI:** 10.3390/audiolres16050138

**Published:** 2026-09-18

**Authors:** Marco Boldreghini, Luigi Panio, Francesca Dominici, Maria Gragnano, Andrea Albera, Claudia Cassandro, Andrea Canale

**Affiliations:** 1 Otoneurophysiology and Biomechanics of Human Movement Laboratory, Department of Surgical Sciences, University of Turin, 10124 Turin, Italy; marco.boldreghini@unito.it; 2 Department of Surgical Sciences, University of Turin, 10126 Turin, Italy; francesca.dominici@unito.it (F.D.); maria.gragnano@unito.it (M.G.); a.albera@unito.it (A.A.); claudia.cassandro@unito.it (C.C.); andrea.canale@unito.it (A.C.)

**Keywords:** cochlear implant, teleaudiology, Remote Check, remote monitoring, impedance telemetry

## Abstract

**Introduction**: Cochlear implant follow-up requires periodic assessments and resources. Remote Check (RC) enables remote assessment of aided thresholds, speech perception in noise, and Impedance Field Telemetry (IFT). This exploratory study investigated relationships between remote and conventional measurements, including numerical and longitudinal comparisons where methodologically appropriate. **Methods**: This prospective, single-center exploratory study included 12 adults with Nucleus 7/8 cochlear implants. At baseline (T0) and after a mean 4.83-month follow-up (T1), participants underwent free-field (FF) pure-tone audiometry, Matrix Sentence Test (MST), Aided Threshold Test (ATT), Digit Triplet Test (DTT), and IFT; RC assessments were performed under supervised clinical conditions. Analyses included exploratory paired comparisons, correlations, longitudinal analyses, and linear mixed-effects models where appropriate. **Results**: ATT thresholds were lower than FF thresholds (mean PTA differences: −7.78 dB at T0; −9.23 dB at T1), with no Method × Time interaction. MST and DTT showed a positive association at T0 (Pearson r = 0.833, 95% CI 0.497–0.952), whereas no clear association was observed at T1 (r = 0.074, 95% CI −0.583–0.672). IFT differences were small (T0: +0.35 kΩ; T1: −0.06 kΩ), with high correlations and no Method × Time interaction. **Discussion and Conclusions**: Under supervised conditions, RC ATT showed numerical differences from conventional FF thresholds, while the relationship between MST and DTT speech-in-noise performance remained uncertain across assessments and remote and conventional IFT measurements showed comparatively small differences. Interpretation is limited by differing measurement paradigms and the small sample. Larger independent studies, including home-based assessments, are required before broader clinical or triage applications can be established.

## 1. Introduction

Cochlear implants are the treatment of choice for severe-to-profound hearing loss that benefits only partially or not at all from traditional hearing amplification, as indicated by the latest Italian guidelines [1].

In Italy, 1746 hospital admissions for cochlear implant surgery were recorded in 2024 across specialized centers [2], with structured long-term follow-up protocols including regular audiological assessment, speech perception testing and device telemetry monitoring [3].

With the growing number of cochlear implant recipients, long-term follow-up has become increasingly demanding in terms of clinical workload, organizational resources, and patient commitment. Teleaudiology encompasses several distinct approaches, including remote programming, asynchronous remote monitoring, home-based self-testing, supervised digital testing, and tele-triage. These approaches differ in their objectives, technical requirements, and level of clinical supervision [4]. In this context, digital monitoring tools may complement conventional in-person follow-up by providing additional information between scheduled clinical assessments.

These tools have not yet been implemented in the routine clinical practice of our center. Before their broader clinical adoption, their measurements and potential limitations should be characterized in relation to conventional clinical assessments, while considering differences in test administration, stimulus presentation, and measurement scales.

This study evaluated Cochlear’s Remote Check, a telemonitoring tool that enables remote assessment of aided thresholds, speech perception in noise, impedance telemetry, and external processor positioning through photographic documentation. The Remote Check assessment includes the Aided Threshold Test (ATT), the Digit Triplet Test (DTT), questionnaires, and impedance telemetry. This system has been described in several studies as a potentially useful tool in the follow-up of patients with cochlear implants, showing good correlation with standard measurements, while highlighting numerical differences that require further investigation. This evidence supports a complementary role for Remote Check, integrating rather than replacing in-person assessment [5,6,7].

Previous Italian studies have investigated Remote Check in cochlear implant recipients [6,7]; however, evidence remains limited regarding the quantitative relationships between Remote Check outcomes and conventional clinical measurements, particularly when different testing paradigms are used.

The primary objective of this exploratory study was to characterize the relationship between Remote Check aided thresholds and conventional free field thresholds. Secondary exploratory objectives included assessment of the association between speech-in-noise performance obtained using the DTT and MST and comparison of impedance telemetry measurements. Because ATT and free-field audiometry, as well as DTT and MST, differ in stimulus presentation and measurement procedures, these comparisons were not intended to establish direct methodological equivalence.

## 2. Materials and Methods

This prospective, single-center exploratory study investigated the relationships and longitudinal changes between measurements obtained using a telemonitoring platform and conventional clinical assessments in cochlear implant recipients. The study was not designed as a clinical equivalence or formal validation study. Assessments were performed at two predefined timepoints. T0 corresponded to the baseline assessment performed at study enrollment, whereas T1 represented the follow-up assessment after a mean interval of 4.83 months. The same clinical and digital assessment protocol was applied at both visits.

The assessments were carried out at the ENT Department of the AOU Città della Salute e della Scienza in Turin, in compliance with current privacy regulations, in accordance with the Declaration of Helsinki [8] and after approval by the ethics committee (Territorial Ethics Committee AOU Città della Salute e della Scienza of Turin, protocol code 120000142, 3 October 2024). The study protocol was not prospectively registered.

The Cochlear™ Remote Check (RC) platform, provided by Cochlear Limited (Sydney, Australia), was used through dedicated study accounts rather than participants’ personal accounts. A dedicated ID and email address were created for each participant. All Remote Check assessments were performed at the hospital using institutional devices rather than participants’ personal devices. This procedure was adopted to standardize testing conditions, provide immediate technical support, and limit the use of participants’ personal identifiers during study procedures.

### 2.1. Statistics

Statistical analyses were performed using ‘R’ software (version 4.5.0; R Foundation for Statistical Computing, Vienna, Austria). Continuous variables were summarized using appropriate descriptive statistics.

For exploratory paired comparisons, numerical differences were defined as the Remote Check measurements minus the corresponding conventional clinical measurement (RC − clinical): ATT − FF for pure-tone thresholds, RC − PC for impedance telemetry. For ATT − FF, numerical differences were considered exploratory and were not interpreted as measurement bias or agreement because ATT and conventional aided free-field audiometry represent distinct measurement procedures and were not assumed to yield interchangeable behavioral thresholds. At each timepoint, paired differences were summarized using the mean, standard deviation, and 95% confidence interval (95% CI). Given the small sample size, mean paired differences were additionally accompanied by bias-corrected and accelerated (BCa) bootstrap 95% CIs based on 10,000 resamples [9]. The distribution of paired differences was explored using the Shapiro–Wilk test; however, selection of the primary analysis was not based solely on formal normality testing. Mean paired differences were tested against zero using paired *t*-tests, with Wilcoxon signed-rank tests performed as sensitivity analyses to assess the robustness of the results to distributional assumptions. For Wilcoxon signed-rank sensitivity analyses, zero differences were omitted and tied absolute differences were assigned average ranks; *p*-values were calculated using the normal approximation with continuity correction rather than exact inference. For ATT − FF, these tests were considered exploratory comparisons of the observed numerical values and were not interpreted as tests of measurement bias, agreement, or equivalence. For frequency-specific pure-tone comparisons, *p*-values were adjusted for multiple testing using the Holm method. Linear associations between remote and conventional clinical measurements for pure-tone thresholds and impedance telemetry were assessed using Pearson’s correlation coefficient (r) with 95% CIs. Correlation was interpreted as a measure of association and not as evidence of agreement.

For speech-in-noise outcomes, MST and DTT SRTs were summarized separately at each timepoint. Their association was explored using Pearson’s correlation coefficient (r) with 95% CIs. Because of the small number of paired observations, Spearman’s rank correlation coefficient (ρ) was additionally calculated as a sensitivity analysis. Correlation analyses were interpreted as measures of association between individual performances and not as evidence of agreement, equivalence, or interchangeability between the two tests. No hypothesis test of the numerical DTT − MST difference was performed.

Bland–Altman analysis [10] was restricted to impedance telemetry, for which RC and PC measurements represent the same underlying quantity on the same measurement scale. Mean differences and 95% limits of agreement were calculated.

Longitudinal analyses were performed to investigate whether the numerical differences between remote and conventional measurements changed from T0 to T1 for pure-tone thresholds and impedance telemetry. Among participants with complete paired observations at both timepoints, within-subject changes were calculated as (RC − clinical) T1 − (RC − clinical) T0 and evaluated using one-sample *t*-tests against zero, with Wilcoxon signed-rank tests as sensitivity analyses and BCa bootstrap 95% CIs based on 10,000 resamples.

In addition, linear mixed-effects models were fitted to account for repeated measurements within participants and to use all available observations. Models included measurement Method, Time (T0/T1), and their interaction as fixed effects, with a participant-specific random intercept. The Method × Time interaction assessed whether the difference between measurements changed over time. Separate models were fitted for PTA, individual pure-tone frequencies, and impedance telemetry. For frequency-specific pure-tone models, interaction *p*-values were adjusted using the Holm method. Degrees of freedom and *p*-values for fixed effects were estimated using Satterthwaite’s approximation as implemented in the lmerTest package (version 3.2.1) [11].

Potentially influential observations identified during graphical and numerical inspection were explored in sensitivity analyses by repeating the relevant analyses after exclusion of the observation and comparing the results with the primary analyses retaining all available data.

Analyses were performed using available observations without imputation. Statistical tests were two-sided, with a significance level of α = 0.05. All procedures were implemented using dedicated scripts developed in R.

### 2.2. Patients

The study sample consisted of 12 adult patients with unilateral cochlear implants, implanted at the AOU Città della Salute e della Scienza in Turin at least 6 months prior. Participants were enrolled according to the following inclusion criteria:

- Age > 18 and <65 years;
- Anacusia or severe/profound sensorineural hearing loss, undergoing surgery for unilateral cochlear implant placement
- Minimum 6 months of cochlear implant use;
- Cochlear Nucleus N7 or N8 processor;
- Free-field speech audiometry with CI turned on, in quiet, ≥60%.
- Free-field speech audiometry with CI turned on, with S0N0 noise ≥ 50% with SNR ≤ +5 (or Matrix Sentence Test S0N0 ≤ +5);
- Complete insertion of the Array with at least 20 active channels;
- Minimum daily use of the processor according to data log: 8 h per day.

The following exclusion criteria were also considered:

- Chronic otitis media with otorrhea;
- Cochlear malformations;
- Central or peripheral degenerative neuropathy;
- Atresia auris or other malformations of the auricle or external auditory canal
- Pediatric patients (age < 18 years).

In participants using bimodal stimulation, the contralateral hearing aid was removed during testing. The contralateral ear was not occluded or masked. Complete contralateral audiometric data were not retrospectively available for all participants; therefore, a contribution of residual acoustic hearing to free-field measurements cannot be entirely excluded. Participants were selected among cochlear implant recipients attending the study center who fulfilled the predefined eligibility criteria. Participants were recruited between March and November 2025. Sampling was non-consecutive. The total number of patients assessed for eligibility and the reasons for non-enrollment were not systematically recorded.

Participation in the study did not result in any changes to routine clinical follow-up or to the usual assessment of cochlear implant functioning.

### 2.3. Measurements

#### 2.3.1. Conventional Audiometry

Conventional audiological measurements (pure-tone audiometry and Matrix Test) were performed by the Audiometric Technician at the ENT Department of the AOU Città della Salute e della Scienza in Turin using a HARP audiometer (Inventis S.r.l., Padua, Italy) routinely used at the study center. The audiometric system underwent the routine calibration procedures used at the study center; the specific calibration record applicable to the study sessions was not retained within the research dataset.

The frequencies tested in the pure-tone audiometry were 250, 500, 1000, 2000, 3000, 4000, 6000, and 8000 Hz. Speech perception in noise was assessed using the Italian Matrix Sentence Test in an open-set condition, introduced and validated in 2015 [12], commonly used in our outpatient setting. The MST was administered using the routine clinical implementation available at the study center. Detailed software-controlled parameters of the adaptive procedure were not separately recorded in the research dataset.

Pure-tone thresholds were obtained according to the routine clinical audiometric procedure used at the study center. Detailed information on the specific stimulus type, step size, and threshold-seeking sequence was not prospectively recorded in the research dataset and could not be reliably reconstructed retrospectively.

Both tests described above were performed in a free field with the cochlear implant turned on, the patient seated in the middle of the room, and two loudspeakers positioned at a 45-degree angle and 1 m away from the subject.

This configuration represents the conventional clinical free-field setup used at our center. This configuration was not intended to reproduce the Remote Check stimulus-delivery pathway and represents an additional methodological difference between the two assessments. Although both conventional aided free-field and ATT thresholds are expressed in dB HL, the two assessments differ in stimulus-delivery pathway, threshold-seeking procedure, and test conditions and were therefore not assumed to yield interchangeable behavioral thresholds.

Individual sound-processor and preprocessing settings were not prospectively standardized as part of the study protocol.

#### 2.3.2. Digital Audiometry

Digital audiometric measurements were performed using Remote Check (Cochlear Limited, Sydney, Australia) at both enrollment and follow-up. All assessments were conducted at the study center under supervised clinical conditions. RC is a tool provided by Cochlear to all cochlear implant users with a Nucleus 7 or Nucleus 8 processor at the time of implant activation, regardless of whether or not they are included in this study.

To ensure standardized execution of procedures, protection of sensitive data, and adequate technical support, all Remote Check sessions were conducted at the center using hospital devices and dedicated accounts.

Since the study only included two RC sessions per patient (T0 and T1), performing them in person also reduced the risk of errors related to the usability of the application, ensuring procedural uniformity.

The application was used to perform the Aided Threshold Test (ATT) at 250, 500, 1000, 2000, 3000, 4000, and 6000 Hz, as well as the Italian Digit Triplet Test (DTT) for adaptive speech-in-noise assessment. ATT results are displayed by the Remote Check platform in dB HL. According to the published technical description of Remote Check, ATT calibration was established at design time by determining frequency-specific multiplication factors for the digital tones to provide the appropriate sound-booth-equivalent dB HL presentation levels. Calibration was verified by ensuring that signals streamed to the sound processor via Bluetooth matched the expected processor output when the same signals were presented acoustically through loudspeakers in an anechoic test box. During ATT and DTT, the sound processor operates in a dedicated Hearing Test mode in which Bluetooth volume control is disabled, microphone input is muted, accessory mixing is set to 100% streaming, and input-processing features are temporarily disabled [5].

However, calibration of ATT in dB HL does not imply that streamed ATT and conventional aided free-field audiometry yield interchangeable behavioral thresholds.

For DTT, the streamed calibration signal is similarly scaled by a global adjustment to ensure the intended presentation level, with calibration verified using the same streamed-versus-acoustic processor-output approach [5].

Within Remote Check, the DTT is an adaptive self-test in which digit triplets are presented in noise and participants select the perceived digits on the application interface. Test stimuli are delivered directly to the sound processor through the Remote Check streaming pathway [5,6]. The signal-to-noise ratio (SNR) is adaptively adjusted according to the participant’s responses, while the combined speech-and-noise presentation level is maintained at 65 dB SPL [13]. The test mode starts at −6 dB SNR and the SNR is subsequently adjusted in ±2 dB steps according to the participant’s responses [5,13]. Following the first reversal, two sets of eight digit triplets are presented, and the mean SNR across the 16 triplets is reported as the speech reception threshold (SRT) [5,6,13]. Although testing was performed under supervised clinical conditions, participants completed the DTT through the RC interface without operator-assisted response selection.

#### 2.3.3. Comparison Between Conventional Free-Field Thresholds and Remote Check ATT Thresholds

Free-field (FF) thresholds were descriptively compared with thresholds obtained using the Remote Check Aided Threshold Test (ATT) at corresponding frequencies from 250 to 6000 Hz at T0 and T1. Although ATT is calibrated to provide sound-booth-equivalent dB HL presentation levels, streamed ATT and conventional aided free-field audiometry differ in signal-delivery pathway, threshold-seeking procedure, test environment, and, in the present study, degree of standardization of processor and acoustic test conditions. The comparison was therefore considered exploratory and was not interpreted as a formal assessment of measurement agreement or interchangeability.

Differences were consistently calculated as ATT − FF. Comparisons were performed for individual frequencies and PTA (PTA4, calculated as the average of thresholds at 500, 1000, 2000, and 4000 Hz), and their longitudinal evolution between T0 and T1 was investigated using within-subject and mixed-effects analyses as described in Section 2.1.

These differences were treated as exploratory numerical comparisons and not as estimates of measurement bias or agreement.

#### 2.3.4. Speech Test Association

Speech perception in noise was assessed using the Italian Matrix Sentence Test (MST) in free field and the Digit Triplet Test (DTT) delivered via Remote Check. Although both outcomes are expressed as speech reception thresholds in dB SNR, MST and DTT differ substantially in speech material, acoustic-phonetic structure, temporal characteristics, lexical content, and signal presentation. Their absolute SRT values were therefore not assumed to be directly interchangeable or to represent equivalent measurements.

The analysis was intended to explore the association between individual performances obtained with the conventional clinical and Remote Check speech-in-noise paradigms rather than to compare their absolute SRT values or establish methodological equivalence. MST and DTT results were therefore summarized separately at each timepoint, and their relationship was assessed using correlation analyses as described in Section 2.1.

#### 2.3.5. IFT Comparison

Impedance Field Telemetry (IFT) was recorded under two conditions: conventional clinical measurement using Custom Sound Pro (Cochlear™, Sydney, Australia) and measurement via Remote Check (RC). For consistency throughout the manuscript, conventional IFT measurements obtained using Custom Sound Pro are referred to as PC measurements. For each subject and timepoint, mean array impedance (kΩ) was calculated as the average of available electrode impedances.

Differences were consistently calculated as RC − PC. Because RC and PC impedance measurements represent the same underlying quantity on the same measurement scale, both association and agreement were evaluated, including Bland–Altman analysis. Longitudinal changes between T0 and T1 were additionally investigated using within-subject and mixed-effects analyses as described in Section 2.1. Analyses were based on available observations, with no imputation of missing values.

## 3. Results

### 3.1. Participants

Twelve adult cochlear implant recipients were included in the study (9 females and 3 males), with a mean age at enrollment of 43.00 ± 13.83 years (range 20–64 years). Mean cochlear implant use at enrollment was 73.67 ± 66.84 months (range 6–198 months), and the mean interval between T0 and T1 was 4.83 ± 1.40 months (158.00 ± 44.56 days). Daily processor use, available for nine participants, averaged 13.78 ± 2.82 h/day. Detailed demographic, clinical, and device characteristics, including median and interquartile range for continuous variables, are reported in Table 1.

Regarding data availability, FF and RC pure-tone threshold measurements were available for most participants at both timepoints. At T0, one ATT measurement at 4000 Hz was unavailable. At T1, ATT measurements were unavailable for one participant at all frequencies and for an additional participant from 2000 to 6000 Hz. Complete MST–DTT pairs were available for all 12 participants at T0 and for 10 participants at T1. Mean IFT values were available for 11 participants at T0 and 10 at T1. Among participants with available IFT measurements, all 22 electrode contacts were reported as active by the platform, and no out-of-range impedance values were observed. All participants attended both scheduled assessments; thus, missing measurements reflected unavailable individual test recordings rather than participant dropout. No substantial deviations from the planned study procedures occurred. Missing measurements were attributable to unavailable or incomplete test recordings and were not considered protocol deviations.

### 3.2. Pure-Tone Threshold: Free Field vs. Remote Check

Remote Check ATT thresholds were generally numerically lower than conventional free-field (FF) pure-tone thresholds across the frequencies investigated at both timepoints. Because ATT and FF represent distinct threshold-measurement procedures, these differences were interpreted as exploratory numerical differences rather than as estimates of measurement bias or formal agreement.

#### 3.2.1. Frequency-Specific Comparison at T0 and T1

**Figure 1:**
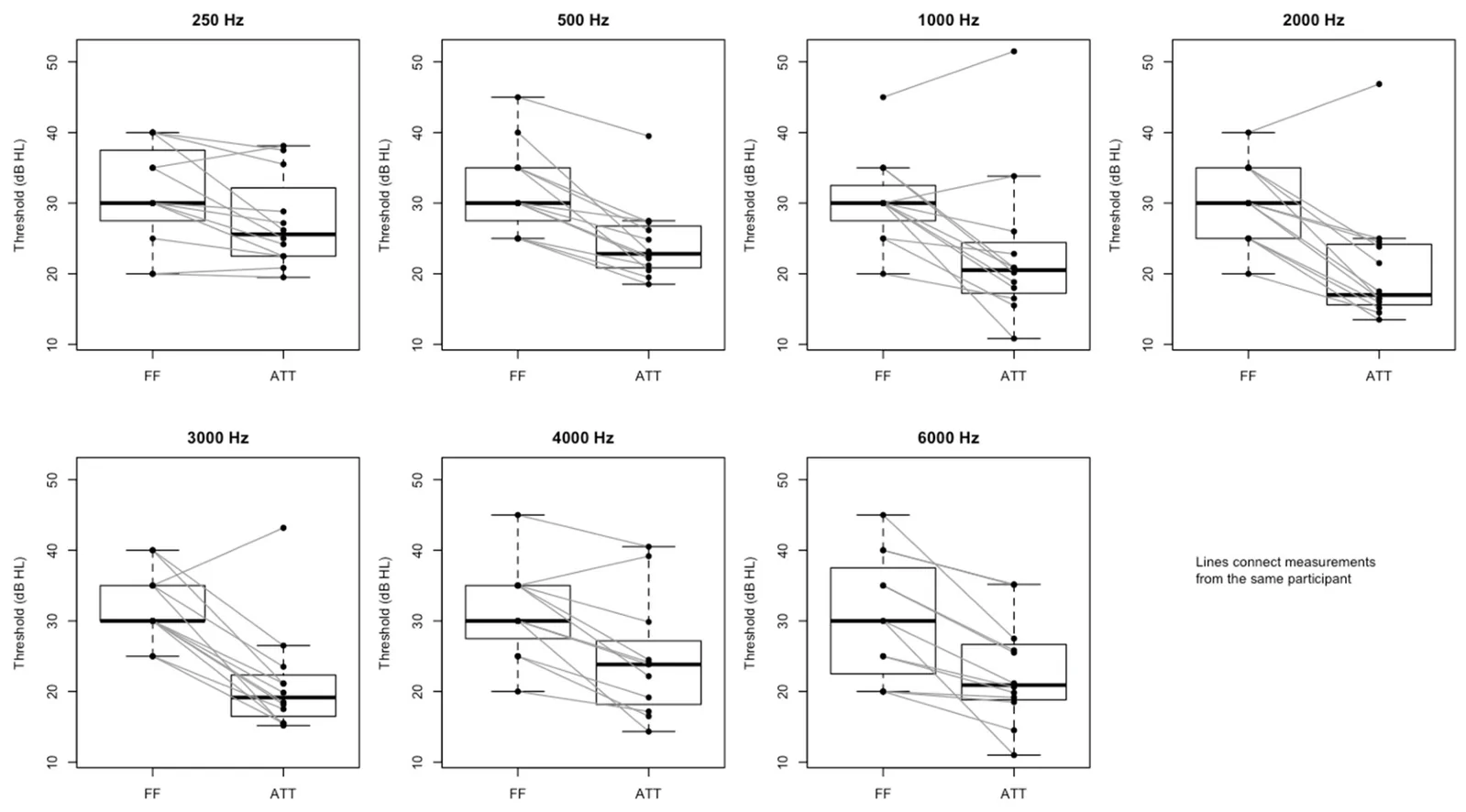
Paired conventional free-field (FF) and Remote Check Aided Threshold Test (ATT) thresholds at T0 across the tested frequencies.

At T0, Remote Check Aided Threshold Test (ATT) thresholds were numerically lower than conventional free-field (FF) thresholds across all tested frequencies. Mean paired differences (ATT − FF) ranged from −3.93 dB HL at 250 Hz to −10.78 dB HL at 3000 Hz. After Holm correction for multiple comparisons, differences remained statistically significant at all frequencies (adjusted *p* ≤ 0.015). BCa bootstrap 95% confidence intervals were entirely below zero at all frequencies. Individual paired FF and ATT measurements at T0 are shown in Figure 1.

**Figure 2:**
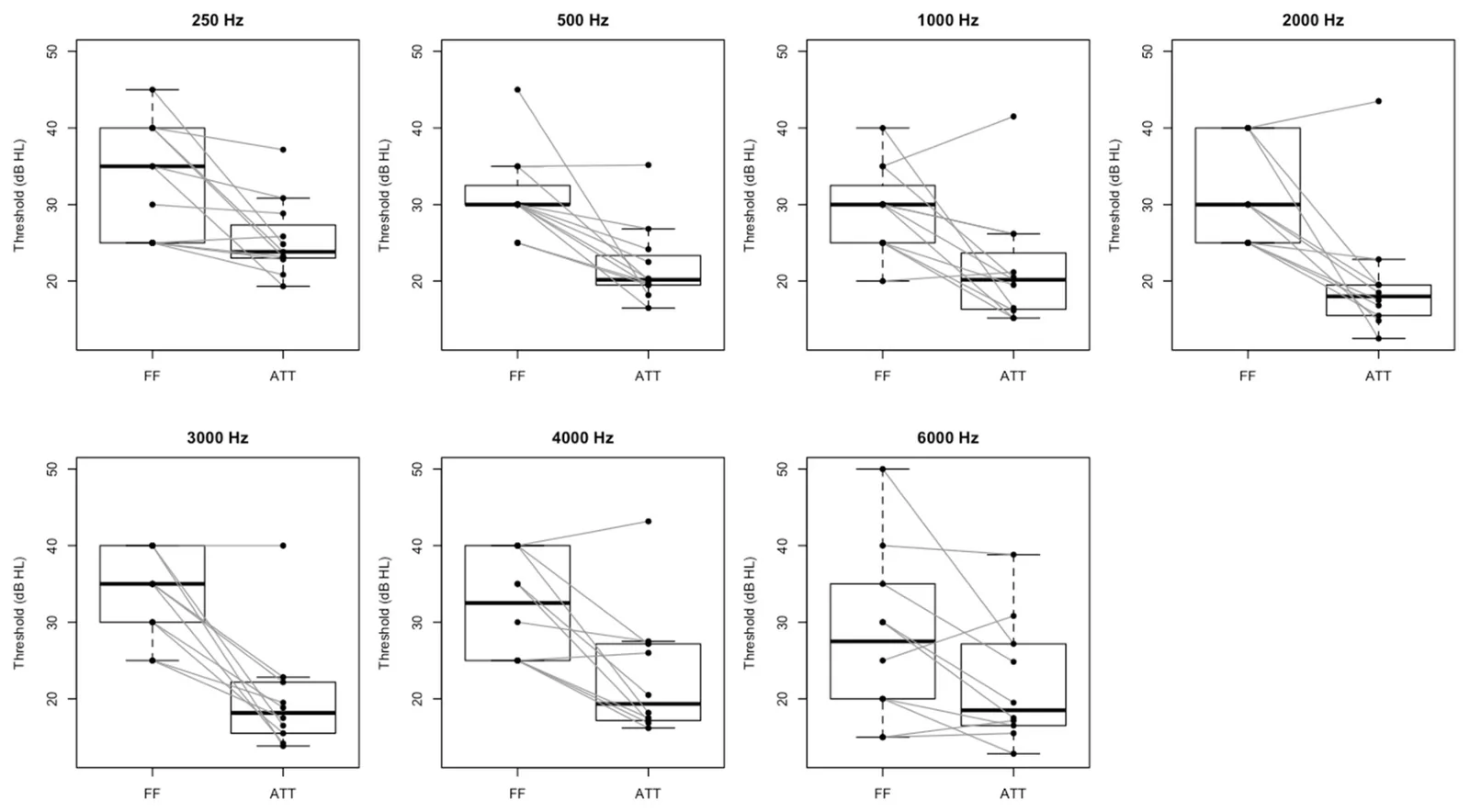
Paired conventional free-field (FF) and Remote Check ATT thresholds at T1 across tested frequencies. Grey solid lines connect measurements obtained from the same participant.

**Figure 3:**
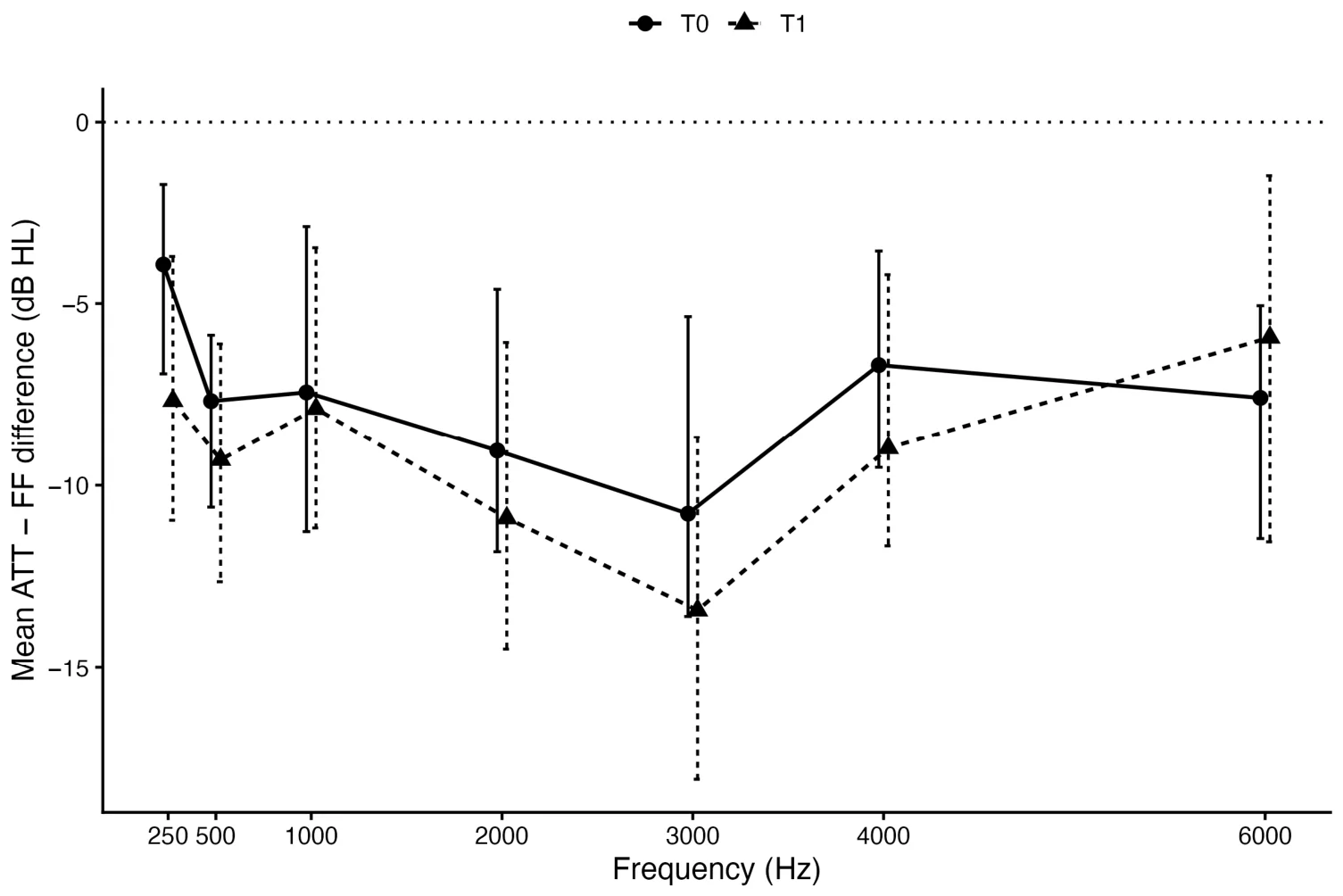
Frequency-specific differences between Aided Threshold Test (ATT) and free-field (FF) pure-tone thresholds at T0 and T1. Points represent the mean paired difference (ATT − FF) at each frequency; error bars indicate bias-corrected and accelerated (BCa) bootstrap 95% confidence intervals. Negative values indicate lower thresholds measured with ATT than with FF.

At T1, ATT thresholds remained numerically lower than FF thresholds across all tested frequencies, with mean paired differences ranging from −5.93 dB HL at 6000 Hz to −13.42 dB HL at 3000 Hz. After Holm correction, differences were statistically significant at six of the seven frequencies (adjusted *p* ≤ 0.025), whereas the difference at 6000 Hz did not reach statistical significance (adjusted *p* = 0.054). BCa bootstrap 95% confidence intervals were below zero at all frequencies. Individual paired measurements at T1 are shown in Figure 2. Frequency-specific mean ATT − FF differences at both timepoints are summarized in Figure 3 and Table 2.

Frequency-specific linear mixed-effects models showed no statistically significant Method × Time interactions at any tested frequency after Holm adjustment (all adjusted *p* = 1.000), providing no evidence that the observed numerical ATT − FF difference changed from T0 to T1 at individual frequencies. For PTA, the mean ATT − FF difference was −7.78 dB HL at T0 (95% CI −10.21 to −5.34; BCa 95% CI −9.56 to −5.34) and −9.23 dB HL at T1 (95% CI −14.36 to −4.10; BCa 95% CI −14.12 to −5.50).

#### 3.2.2. Comparison Between ATT and FF Pure-Tone Averages

Pure-tone averages (PTA4; 500, 1000, 2000, and 4000 Hz) were compared between ATT and FF measurements. At T0, mean PTA was 30.83 dB HL for FF and 23.06 dB HL for ATT, corresponding to a mean ATT − FF difference of −7.78 dB (95% CI −10.21 to −5.34; *p* < 0.001). At T1, mean PTA was 30.68 dB HL for FF and 21.45 dB HL for ATT, with a mean difference of −9.23 dB (95% CI −14.36 to −4.10; *p* = 0.002). Thus, ATT yielded numerically lower PTA thresholds than conventional FF testing at both timepoints. BCa bootstrap 95% CIs were consistent with these findings (T0: −9.56 to −5.34 dB; T1: −14.12 to −5.50 dB).

Among the 11 participants with complete PTA measurements at both timepoints, the mean within-subject change in the ATT − FF difference from T0 to T1 was −1.19 dB (95% CI −5.85 to 3.47; *p* = 0.582), indicating no significant longitudinal change in the magnitude of the difference. A Wilcoxon signed-rank sensitivity analysis yielded consistent results (*p* = 0.756).

In the linear mixed-effects model, the Method × Time interaction was not statistically significant (β = −1.40 dB, 95% CI −5.79 to 2.98; *p* = 0.535), providing no evidence of a change in the observed numerical difference between ATT and FF measurements from T0 to T1.

#### 3.2.3. Association Between ATT and FF Thresholds

At T0, moderate-to-strong positive correlations were observed at most frequencies, with statistically significant associations at all frequencies except 3000 Hz. At T1, correlations were generally weaker and more variable, with a statistically significant association observed only at 6000 Hz. PTA showed a strong correlation at T0 (r = 0.873, 95% CI 0.601 to 0.964; *p* < 0.001), whereas the correlation was weaker and not statistically significant at T1 (r = 0.356, 95% CI −0.310 to 0.788; *p* = 0.283) (Table 3). These correlations describe the linear association between ATT and FF thresholds and should not be interpreted as measures of agreement.

### 3.3. Speech-in-Noise Performance: Association Between Matrix Sentence Test and Digit Triplet Test

At T0, 12 paired observations were available. Mean speech reception threshold was 6.04 dB SNR (SD 4.68) for the Matrix Sentence Test and −0.28 dB SNR (SD 4.47) for the DTT. A positive association was observed between MST and DTT thresholds (Pearson r = 0.833, 95% CI 0.497 to 0.952; *p* < 0.001). The rank-based sensitivity analysis also showed a positive, although more moderate, association (Spearman ρ = 0.599; *p* = 0.040).

At T1, 10 paired observations were available. Mean thresholds were 4.59 dB SNR (SD 5.33) for MST and −1.92 dB SNR (SD 3.30) for DTT. No clear association was observed between MST and DTT thresholds (Pearson r = 0.074, 95% CI −0.583 to 0.672; *p* = 0.840), with similar findings in the rank-based sensitivity analysis (Spearman ρ = 0.109; *p* = 0.763).

**Figure 4:**
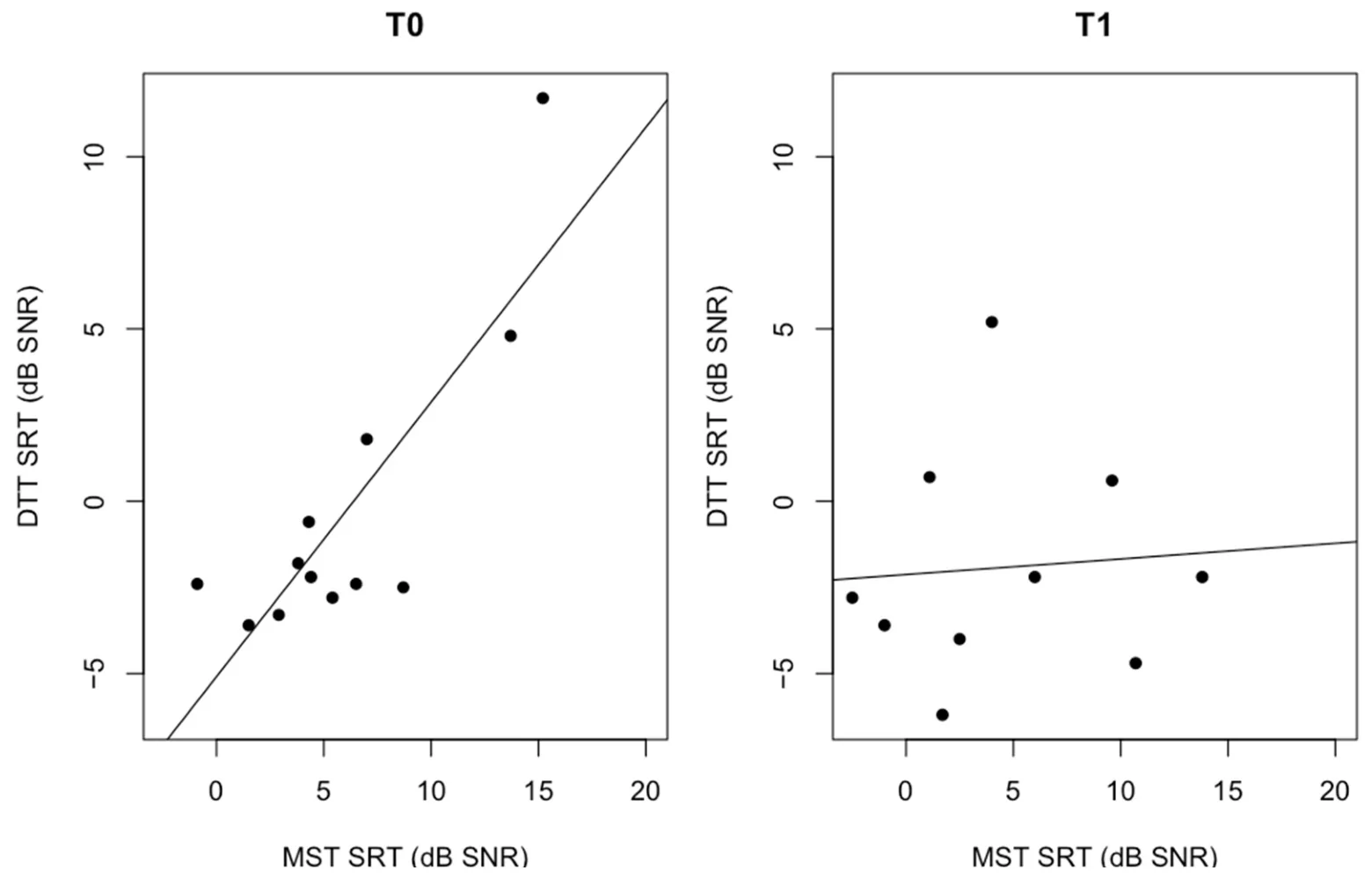
Association between MST and DTT speech reception thresholds at T0 and T1. Each point represents one participant. Solid lines represent the fitted linear regression at each timepoint.

Given the small number of paired observations, particularly at T1, these estimates should be interpreted cautiously. Individual MST and DTT measurements and their associations at T0 and T1 are shown in Figure 4, while descriptive and correlation results are summarized in Table 4.

### 3.4. Impedance Field Telemetry

Impedance Field Telemetry measurements obtained through Remote Check showed small mean differences from conventional PC measurements at both timepoints. At T0 (*n* = 11), mean impedance was 8.02 kΩ with PC and 8.37 kΩ with RC, corresponding to a mean RC − PC difference of 0.35 kΩ (95% CI −0.24 to 0.95; BCa 95% CI 0.01 to 1.15; *p* = 0.217). At T1 (*n* = 10), mean impedance was 7.84 kΩ with PC and 7.78 kΩ with RC, with a mean difference of −0.06 kΩ (95% CI −0.41 to 0.29; BCa 95% CI −0.44 to 0.16; *p* = 0.700).

Descriptive, comparative, and association results are summarized in Table 5.

#### 3.4.1. Agreement and Association Between RC and PC

Bland–Altman analysis showed 95% limits of agreement from −1.38 to 2.09 kΩ at T0 and from −1.01 to 0.89 kΩ at T1. Pearson correlations between RC and PC measurements were strong at both timepoints (T0: r = 0.920, 95% CI 0.715 to 0.979; *p* < 0.001; T1: r = 0.969, 95% CI 0.870 to 0.993; *p* < 0.001).

**Figure 5:**
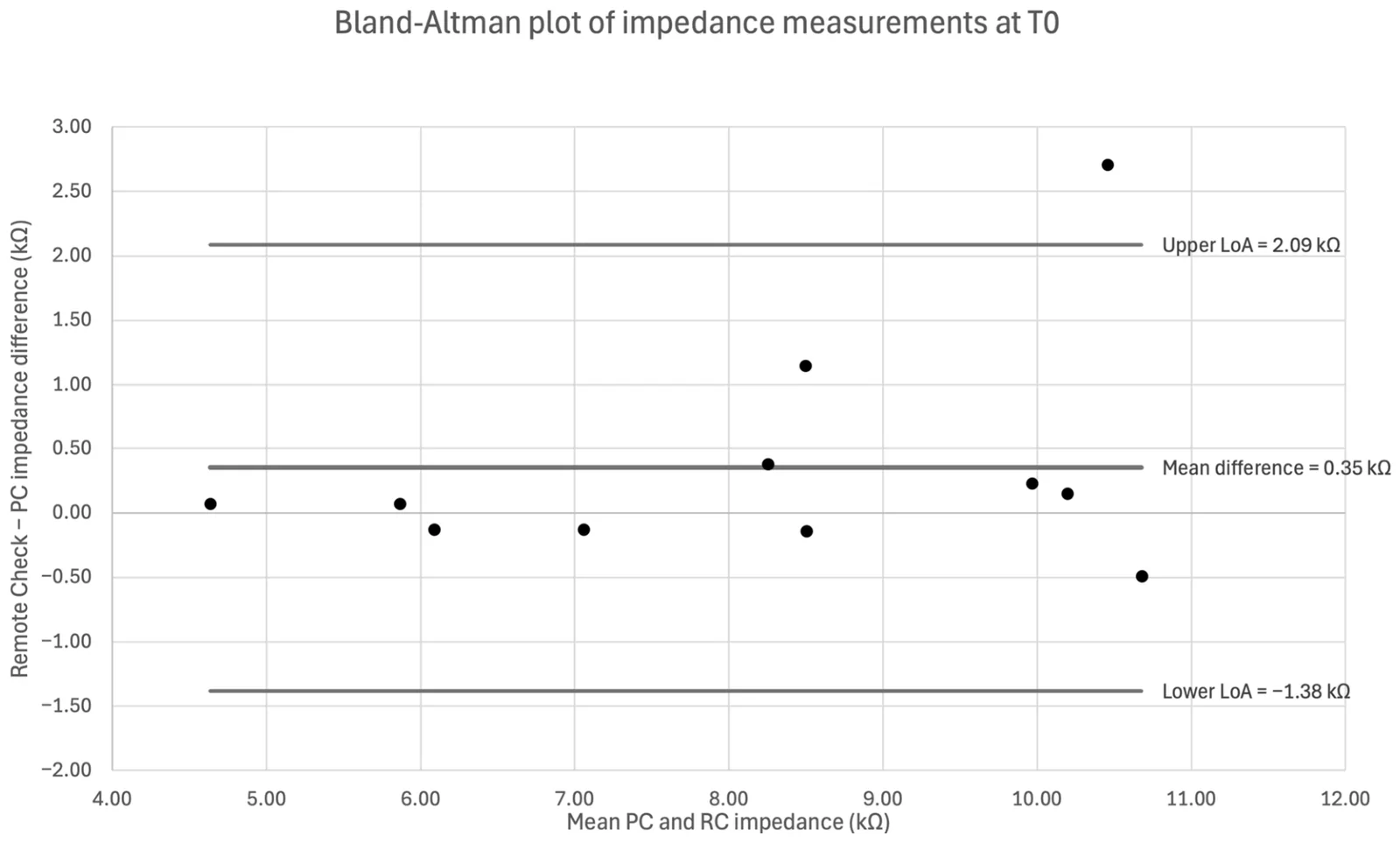
Bland–Altman plot comparing Remote Check (RC) and conventional programming software (PC) impedance measurements at T0. The solid central line represents the mean RC − PC difference, and the upper and lower lines represent the 95% limits of agreement (mean difference ± 1.96 SD). Each point represents one participant.

**Figure 6:**
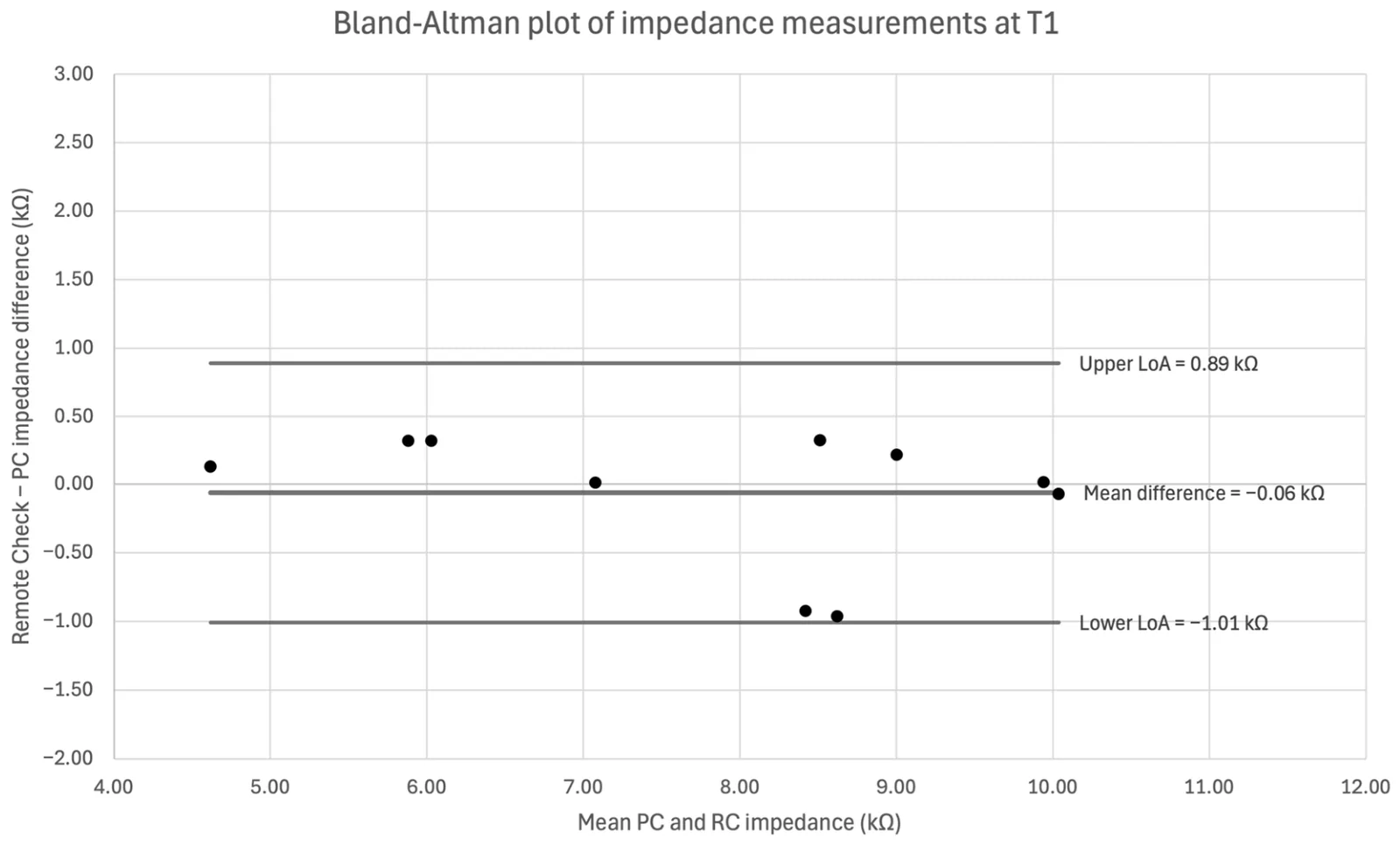
Bland–Altman plot comparing Remote Check (RC) and conventional programming software (PC) impedance measurements at T1. The solid central line represents the mean RC − PC difference, and the upper and lower lines represent the 95% limits of agreement (mean difference ± 1.96 SD). Each point represents one participant.

The Bland–Altman plots illustrating the distribution of individual RC − PC differences and the corresponding 95% limits of agreement at T0 and T1 are shown in Figure 5 and Figure 6, respectively.

#### 3.4.2. Longitudinal Analysis

Among participants with measurements available at both timepoints, the mean within-subject change in the RC − PC difference from T0 to T1 was −0.50 kΩ (95% CI −1.27 to 0.27; BCa 95% CI −1.26 to 0.03; *p* = 0.178). The Wilcoxon signed-rank sensitivity analysis yielded consistent results (*p* = 0.541).

Consistently, the Method × Time interaction in the linear mixed-effects model was not statistically significant (β = −0.41 kΩ, 95% CI −1.02 to 0.20; *p* = 0.194), providing no evidence of a systematic change in the difference between RC and PC impedance measurements over time.

Sensitivity analysis excluding the potentially influential observation yielded a similar conclusion (mean change −0.28 kΩ, 95% CI −0.94 to 0.39; *p* = 0.367).

## 4. Discussion

This exploratory study compared Remote Check outcomes with conventional clinical measurements for aided thresholds, speech perception in noise, and impedance telemetry. The main findings were numerical differences between ATT and free-field thresholds, a positive association between DTT and MST speech reception thresholds at T0 but no clear association at T1, and comparatively small differences between remote and conventional impedance measurements. Because ATT and free-field audiometry differ in their measurement procedures, their numerical differences were not interpreted as formal measurement bias or evidence of equivalence. For speech-in-noise outcomes, DTT and MST were treated as methodologically distinct paradigms, and their relationship was therefore interpreted only in terms of association rather than numerical agreement or interchangeability.

### 4.1. Pure-Tone Thresholds: Differences Between ATT and Free-Field Measurements

ATT thresholds were lower than conventional free-field thresholds at both timepoints. At the PTA level, mean differences were −7.78 dB at T0 and −9.23 dB at T1. The Method × Time interaction was not statistically significant, providing no evidence that the numerical difference changed during follow-up.

A difference in the same direction was reported by Maruthurkkara et al. [5], who found streamed ATT thresholds to be, on average, 6.7 dB lower than clinician-administered aided free-field thresholds. Remote Check ATT uses a predefined calibration procedure in which frequency-specific scaling of the digital test tones is used to provide sound-booth-equivalent dB HL presentation levels, with calibration verified against the expected sound-processor output during acoustic presentation [5]. Notably, this difference was observed under more tightly standardized experimental conditions than those used in the present study, including matched processor settings, disabled input-processing features, controlled loudspeaker presentation, and session-specific sound-field calibration. The direction of the difference is consistent with the present findings, with a broadly comparable magnitude.

Nevertheless, calibration of the ATT presentation level does not establish interchangeability between streamed ATT and conventional aided free-field behavioral thresholds, and the present study was not designed to isolate the source of the observed numerical difference. Potential contributors include differences in signal-delivery pathway, threshold-seeking procedures, loudspeaker configuration, room acoustics, test administration, and sound-processor settings.

Accordingly, the observed ATT − FF differences cannot be attributed specifically to calibration and should not be interpreted as evidence that either procedure provides more accurate behavioral thresholds.

Correlations between ATT and free-field thresholds were stronger at T0 and more variable at T1. These correlations describe association between the measurements and should not be interpreted as evidence of agreement or interchangeability.

### 4.2. Speech in Noise

A positive association between MST and DTT speech-in-noise performance was observed at T0, whereas no clear association was observed at T1. Rank-based sensitivity analyses showed the same overall pattern, although the association at T0 was more moderate. These findings should be interpreted cautiously given the small number of paired observations and the resulting uncertainty, particularly at T1.

Although both tests report results in dB SNR, their absolute values should not be considered directly comparable [14,15]. MST and DTT differ in speech material, acoustic-phonetic structure, coarticulation, temporal characteristics, prosody, lexical content, semantic redundancy, response format, and signal delivery [12,15,16]. Regarding signal delivery, MST was performed in free field, whereas DTT was delivered through the Remote Check pathway. Cognitive and linguistic factors may also influence speech-in-noise performance, but they were not directly assessed in the present study.

Accordingly, the observed associations describe the relationship between individual performance on two distinct speech-in-noise paradigms and should not be interpreted as evidence of agreement, equivalence, or interchangeability.

The marked descriptive difference between the T0 and T1 correlation estimates should not be interpreted as evidence of a true change in the relationship between MST and DTT over time. In particular, the wide 95% CI around the T1 Pearson correlation (−0.583 to 0.672) indicates substantial imprecision and is compatible with a broad range of underlying associations. The present findings therefore do not establish whether the relationship between MST and DTT is stable across repeated assessments.

### 4.3. Impedance Field Telemetry: Agreement Between Remote Check and Clinical Measurements

Unlike the ATT–FF and DTT–MST comparisons, Remote Check and conventional IFT measurements represent the same underlying quantity on the same measurement scale. Mean differences were small at both timepoints, with Bland–Altman limits of agreement of −1.38 to 2.09 kΩ at T0 and −1.01 to 0.89 kΩ at T1. Correlations were high at both assessments, and no significant Method × Time interaction was observed. Similar findings have been reported by Schepers et al., who found no significant differences in IFT measurements obtained during local and remote cochlear implant fitting [17]. These findings support further evaluation of Remote Check for longitudinal impedance monitoring, while the limited sample size should be considered.

### 4.4. Limitations and Perspectives

The principal limitations of this study are the small sample size and single-center design, which limit the precision and generalizability of the estimates. Missing measurements further reduced the number of observations available for some comparisons, particularly at T1. Although all participants completed both study visits, these missing recordings reduced the precision of the estimates and should be considered when interpreting the variability of the results. Participants were recruited using non-consecutive sampling, and the total number of potentially eligible patients who were not enrolled was not systematically recorded. Selection bias therefore cannot be excluded.

In participants using bimodal stimulation, the contralateral hearing aid was removed during testing, but the contralateral ear was not occluded or masked. Since complete contralateral audiometric data were not available for all participants, a contribution of residual acoustic hearing to free-field measurements cannot be excluded.

For speech-in-noise assessment, complete MST–DTT pairs were available for all 12 participants at T0 and for 10 participants at T1. The small number of paired observations resulted in limited precision of the correlation estimates, particularly at T1, and should be considered when interpreting the marked descriptive difference between the two timepoints. Missing Remote Check IFT measurements occurred in one participant at T0 and two participants at T1 due to the remote test upload not being completed. These missing measurements further reduced the number of paired IFT comparisons.

A further limitation is the use of different speech-in-noise paradigms in the two assessment modes. Although MST and DTT both provide SRT values in dB SNR, they differ in speech material, test structure, response format, and delivery pathway. Their absolute values should therefore not be considered directly interchangeable, and the association analyses performed in this study should be regarded as exploratory and should not be interpreted as evidence of agreement or equivalence between the two tests.

All Remote Check assessments were performed under supervised clinical conditions rather than at home. The findings therefore cannot be directly generalized to unsupervised home-based use.

Sound-processor settings were not prospectively standardized or documented in sufficient detail for all participants. Their potential contribution to differences between free-field and streamed assessments could therefore not be quantified.

Although ATT has a published calibration procedure designed to provide sound-booth-equivalent dB HL presentation levels, this does not establish interchangeability with conventional aided free-field behavioral thresholds. In the present study, the specific free-field calibration record applicable to the study sessions was not retained within the research dataset, and several procedural parameters and sound-processor settings were not prospectively standardized. These factors limit attribution of the observed ATT − FF differences to any single component of the measurement procedures and preclude their interpretation as a formal calibration offset or measurement bias.

Considering these limitations, future studies should include larger multicenter cohorts and independent samples to assess the reproducibility and generalizability of these findings. Speech-in-noise performance should be compared using matched test paradigms and delivery conditions, including validated Italian digit-in-noise approaches [18], allowing test-related and presentation-related differences to be evaluated separately. Home-based assessments are also needed to determine whether findings obtained under supervised clinical conditions are reproducible in the intended remote setting. These studies would help define the appropriate role of Remote Check within cochlear implant follow-up.

## 5. Conclusions

This exploratory study found lower Remote Check ATT thresholds than conventional aided free-field thresholds. Although ATT has a defined calibration procedure designed to provide sound-booth-equivalent dB HL presentation levels, streamed ATT and conventional aided free-field audiometry represent distinct behavioral measurement procedures and should not be assumed to yield interchangeable thresholds. Accordingly, the observed ATT − FF differences should not be interpreted as formal measurement bias or evidence of equivalence.

Associations between ATT and free-field thresholds varied between timepoints. For speech-in-noise outcomes, a positive association between MST and DTT was observed at T0, whereas no clear association was observed at T1; given the small number of paired observations, these findings do not establish whether the relationship between the two tests is stable across repeated assessments. Remote and conventional impedance measurements showed comparatively small differences and high associations. Given the small, selected sample and the supervised clinical setting, these findings should be considered preliminary. Larger independent studies, including home-based assessments and clearly defined method-comparison protocols, are required before broader clinical or triage applications can be established.

## Figures and Tables

**Table 1:** Demographic, clinical and cochlear implant characteristics of the study population. Data are presented as mean ± standard deviation (SD); median (interquartile range [IQR]); range for continuous variables, and as *n* (%) for categorical variables.

Characteristic	Study Population (*n* = 12)
**Age at enrollment, years**	43.00 ± 13.83; 47.00 (35.00–51.25); 20–64
**Duration of cochlear implant use, months**	73.67 ± 66.84; 74.50 (11.75–109.25); 6–198
**Daily processor use, h/day**	13.78 ± 2.82; 14.00 (13.00–15.00); 9–18
**Sex, *n* (%)**	
Female	9 (75.0)
Male	3 (25.0)
**Onset of hearing loss, *n* (%)**	
Congenital	2 (16.7)
Prelingual	2 (16.7)
Childhood	2 (16.7)
Unknown/not available	6 (50.0)
**Cochlear implant model, *n* (%)**	
CI622	5 (41.7)
CI522	3 (25.0)
CI512	2 (16.7)
CI532	1 (8.3)
CI24Re(Ca)	1 (8.3)
**Electrode array, *n* (%)**	
Slim straight	8 (66.7)
Contour advance	3 (25.0)
Slim modiolar	1 (8.3)
**Sound processor, *n* (%)**	
CP1000	1 (8.3)
CP1110	11 (91.7)
**Implanted side, *n* (%)**	
Right	6 (50.0)
Left	6 (50.0)

**Table 2:** Frequency-specific comparison between conventional free-field (FF) and Remote Check Aided Threshold Test (ATT) thresholds at T0 and T1.

Frequency (Hz)	Timepoint	*n*	Mean FF (dB HL)	Mean ATT (dB HL)	Mean Difference ATT − FF (dB HL)	95% CI	BCa 95% CI	Holm-Adjusted *p*
250	T0	12	31.25	27.32	−3.93	−6.95 to −0.92	−6.93 to −1.71	0.015
500	T0	12	32.08	24.40	−7.68	−10.32 to −5.04	−10.60 to −5.87	<0.001
1000	T0	12	30.42	22.98	−7.44	−12.35 to −2.54	−11.27 to −2.89	0.013
2000	T0	12	30.00	20.95	−9.05	−13.10 to −5.00	−11.82 to −4.61	0.002
3000	T0	12	32.08	21.30	−10.78	−15.26 to −6.30	−13.59 to −5.36	0.002
4000	T0	11	31.36	24.67	−6.69	−10.24 to −3.15	−9.51 to −3.56	0.005
6000	T0	12	30.42	22.82	−7.59	−12.27 to −2.91	−11.46 to −5.06	0.003
250	T1	11	33.18	25.51	−7.67	−12.89 to −2.45	−10.96 to −3.71	0.025
500	T1	11	31.36	22.06	−9.30	−14.49 to −4.11	−12.64 to −6.11	0.010
1000	T1	11	29.55	21.65	−7.89	−13.08 to −2.70	−11.17 to −3.47	0.025
2000	T1	10	31.00	20.10	−10.90	−17.09 to −4.71	−14.48 to −6.07	0.017
3000	T1	10	31.50	18.08	−13.42	−19.81 to −7.02	−18.05 to −8.68	0.004
4000	T1	10	31.00	22.02	−8.98	−14.28 to −3.68	−11.66 to −4.21	0.025
6000	T1	10	31.00	25.07	−5.93	−11.98 to 0.11	−11.55 to −1.47	0.054

Differences were calculated as ATT − FF; negative values indicate lower thresholds with ATT. Values are reported in dB HL. BCa, bias-corrected and accelerated bootstrap confidence interval based on 10,000 resamples. *p*-values refer to paired *t*-tests and were adjusted for multiple frequency-specific comparisons using the Holm method.

**Table 3:** Pearson’s correlation between Aided Threshold Test (ATT) and free-field (FF) thresholds by frequency and timepoint (T0 and T1), including pure-tone average (PTA).

Frequency (Hz)	Timepoint	*n*	r	95% CI	*p*
250	T0	12	0.758	0.326 to 0.928	0.004
500	T0	12	0.756	0.321 to 0.927	0.004
1000	T0	12	0.698	0.206 to 0.908	0.012
2000	T0	12	0.723	0.255 to 0.917	0.008
3000	T0	12	0.446	−0.172 to 0.812	0.147
4000	T0	11	0.795	0.374 to 0.945	0.003
6000	T0	12	0.760	0.331 to 0.929	0.004
PTA	T0	12	0.873	0.601 to 0.964	<0.001
250	T1	11	0.286	−0.379 to 0.756	0.395
500	T1	11	0.076	−0.549 to 0.646	0.825
1000	T1	11	0.270	−0.393 to 0.749	0.421
2000	T1	10	0.370	−0.339 to 0.811	0.293
3000	T1	10	0.279	−0.425 to 0.773	0.435
4000	T1	10	0.453	−0.247 to 0.842	0.189
6000	T1	10	0.670	0.069 to 0.914	0.034
PTA	T1	11	0.356	−0.310 to 0.788	0.283

**Table 4:** Descriptive speech reception thresholds and association between MST and DTT at T0 and T1.

Timepoint	*n*	Mean MST (SD), dB SNR	Mean DTT (SD), dB SNR	Pearson r	Pearson 95% CI	*p*	Spearman ρ	*p*
T0	12	6.04 (4.68)	−0.28 (4.47)	0.833	0.497 to 0.952	<0.001	0.599	0.040
T1	10	4.59 (5.33)	−1.92 (3.30)	0.074	−0.583 to 0.672	0.840	0.109	0.763

Values are reported in dB SNR. Pearson’s correlation coefficient (r) was used as the primary measure of linear association, with Spearman’s rank correlation coefficient (ρ) as a sensitivity analysis. Correlation analyses assess association and should not be interpreted as evidence of agreement, equivalence, or interchangeability between MST and DTT.

**Table 5:** Comparison, agreement, and association between conventional programming software (PC) and Remote Check (RC) impedance measurements at T0 and T1.

Timepoint	*n*	Mean PC (kΩ)	Mean RC (kΩ)	Mean Difference RC − PC (kΩ)	95% CI	BCa 95% CI	95% LoA	Pearson r (95% CI)	Pearson *p*
T0	11	8.02	8.37	0.35	−0.24 to 0.95	0.01 to 1.15	−1.38 to 2.09	0.920 (0.715 to 0.979)	<0.001
T1	10	7.84	7.78	−0.06	−0.41 to 0.29	−0.44 to 0.16	−1.01 to 0.89	0.969 (0.870 to 0.993)	<0.001

Differences were calculated as RC − PC and are reported in kΩ. BCa, bias-corrected and accelerated bootstrap confidence interval based on 10,000 resamples; LoA, limits of agreement. Pearson’s r describes the linear association between PC and RC measurements.

## Data Availability

The data supporting the findings of this study are not publicly available due to privacy and ethical restrictions. Requests for access to the data may be directed to the corresponding author and will be considered in accordance with applicable ethical and privacy requirements.

## Abbreviations

The following abbreviations are used in this manuscript:

ATT	Aided Threshold Test
BCa	Bias-Corrected and Accelerated
CI	Cochlear Implant
DTT	Digit Triplet Test
FF	Free-Field
IFT	Impedance Field Telemetry
IQR	Interquartile Range
LoA	Limits of Agreement
MST	Matrix Sentence Test
PC	Conventional clinical measurement using Custom Sound Pro
PTA	Pure-Tone Average
RC	Remote Check
SD	Standard Deviation
SNR	Signal-to-Noise Ratio
SRT	Speech Reception Threshold
95% CI	95% Confidence Interval
